# Group-sequential analysis may allow for early trial termination: illustration by an intra-observer repeatability study

**DOI:** 10.1186/s13550-017-0328-6

**Published:** 2017-09-26

**Authors:** Oke Gerke, Mie H. Vilstrup, Ulrich Halekoh, Malene Grubbe Hildebrandt, Poul Flemming Høilund-Carlsen

**Affiliations:** 1 Department of Nuclear Medicine, Odense University Hospital, J.B. Winsløws Vej 4, 5000 Odense C, Denmark; 2 Centre of Health Economics Research, University of Southern Denmark, Campusvej 55, 5230 Odense M, Denmark; 3 Epidemiology, Biostatistics and Biodemography, University of Southern Denmark, J.B. Winsløws Vej 9b, 5000 Odense C, Denmark; 4 Department of Clinical Research, University of Southern Denmark, Winsløwparken 19, 5000 Odense C, Denmark

**Keywords:** Agreement, Bland-Altman plot, Repeatability, Reproducibility, Sample size

## Abstract

**Background:**

Group-sequential testing is widely used in pivotal therapeutic, but rarely in diagnostic research, although it may save studies, time, and costs. The purpose of this paper was to demonstrate a group-sequential analysis strategy in an intra-observer study on quantitative FDG-PET/CT measurements, illuminating the possibility of early trial termination which implicates significant potential time and resource savings.

**Methods:**

Primary lesion maximum standardised uptake value (SUVmax) was determined twice from preoperative FDG-PET/CTs in 45 ovarian cancer patients. Differences in SUVmax were assumed to be normally distributed, and sequential one-sided hypothesis tests on the population standard deviation of the differences against a hypothesised value of 1.5 were performed, employing an alpha spending function. The fixed-sample analysis (*N* = 45) was compared with the group-sequential analysis strategies comprising one (at *N* = 23), two (at *N* = 15, 30), or three interim analyses (at *N* = 11, 23, 34), respectively, which were defined post hoc.

**Results:**

When performing interim analyses with one third and two thirds of patients, sufficient agreement could be concluded after the first interim analysis and the final analysis. Other partitions did not suggest early stopping after adjustment for multiple testing due to one influential outlier and our small sample size.

**Conclusions:**

Group-sequential testing may enable early stopping of a trial, allowing for potential time and resource savings. The testing strategy must, though, be defined at the planning stage, and sample sizes must be reasonably large at interim analysis to ensure robustness against single outliers. Group-sequential testing may have a place in accuracy and agreement studies.

**Electronic supplementary material:**

The online version of this article (10.1186/s13550-017-0328-6) contains supplementary material, which is available to authorized users.

## Background

Planning, conduct, analysis, and report of clinical trials require comprehensive resources. Most clinical trials employ fixed-sample designs in which the data of all patients are collected and first examined at the end of the study. In contrast, group-sequential trial designs are hallmarked by predefinition of number, time points, and stopping rules of interim analyses to enable the trial to be terminated early due to either fertility or futility should the trial develop against former expectations. This built-in possibility requires adjustment of analyses for multiple testing, for which suitable approaches are at hand [1, 2]. While group-sequential testing is regularly done in pivotal clinical trials with therapeutic intent, it is much less common in diagnostic trials. The purpose of this paper was to demonstrate a group-sequential analysis strategy in an intra-observer study on quantitative FDG-PET/CT measurements, illuminating the possibility of early trial termination which implicates significant potential time and resource savings.

## Methods

### Bland-Altman limits of agreement

The agreement of paired, quantitative measurements in method comparison or observer variation studies is often assessed with Bland-Altman plots with respective limits of agreement [3, 4]. These limits consist of the mean difference of the paired measurements ± 1.96 times the sample standard deviation of these differences [5–7]. Implicitly, it is assumed that the paired differences of the whole target population from which the sample was taken follow a normal distribution; then, the Bland-Altman limits of agreement comprise, on average, 95% of all observations according to the 68-95-99.7 rule [8] and can be interpreted as prediction interval.

In the following, we will base the statistical analysis strategy on the true (but unknown) population standard deviation of the paired differences. By this means, the Bland-Altman limits of agreement and the applied statistical hypothesis test are by definition interrelated.

### Primary hypothesis

We are interested in testing that the true (but unknown) population standard deviation of the paired differences is smaller than a benchmark below which agreement would be assessed to be acceptable from a clinical point of view. Therefore, our primary hypothesis reads: *The observed sample standard deviation falls sufficiently small of a predefined benchmark, implicating that the true (but unknown) population standard deviation is likely to be smaller than that benchmark as well.* Or, in more technical terms: we will test statistically whether the sample standard deviation is significantly smaller than the benchmark.

### Statistical test

The respective statistical hypothesis test to answer the primary hypothesis above is a one-sided hypothesis test on the population standard deviation of the paired differences between measurements (*σ*) against a predefined benchmark (*σ*_0_):

Null hypothesis (H_0_): *σ* ≥ *σ*_0_ vs. alternative hypothesis (H_a_): *σ* < *σ*_0_.

Assuming that the paired differences follow a normal distribution, the test statistic

$$ {\chi}^2=\frac{\left(n-1\right){s}^2}{{\sigma_o}^2} $$

follows a chi-square distribution with *n* − 1 degrees of freedom, where *n* denotes the number of paired measurements and *s* the sample standard deviation [8, 9]. Corresponding upper one-sided confidence limits are constructed by using the very same test statistic and were supplemented.

Below, we will work with the benchmark *σ*_0_ = 1.5 for exemplification purposes.

### Group-sequential testing with an α-spending function

The spending function approach specifies a sequential design directly in terms of *α*_*t*_, the significance levels for interim and final analyses which depend on the amount of hitherto accumulated information in terms of observations gathered. The basic idea is to use significance levels smaller than the nominal significance level (of usually 5%) in interim analyses in order to secure that the probability of falsely rejecting the null hypothesis at any interim analysis or at the final analysis does not exceed the nominal significance level. We set this nominal experiment-wise level of significance, *α*, to 5% and employed the α-spending function *α*_*t*_ = *αt* [10], where *t* and *α*_*t*_ denote the proportion of accumulated information and the significance level to which the realised *P* value is to be compared with at a particular analysis time point, respectively. Here, we defined post hoc three different group-sequential analysis strategies comprising one (at *N* = 23), two (at *N* = 15, 30), or three interim analyses (at *N* = 11, 23, 34), respectively. These led to significance levels for the interim analyses of *α*_0.5_ = 0.05 × 1/2 = 0.025; *α*_0.33_ = 0.05 × 1/3 = 0.0167 and *α*_0.67_ = 0.05 × 2/3 = 0.0333; and *α*_0.25_ = 0.05 × 1/4 = 0.0125, *α*_0.5_ = 0.05 × 1/2 = 0.025, and *α*_0.75_ = 0.05 × 3/4 = 0.0375, respectively. Final analysis was done with 45 patients and a significance level *α*_1_ = 0.05.

### Clinical example

We used data from an ongoing clinical study in patients with suspicion of ovarian cancer which was described elsewhere [11]. In brief, this study’s primary hypothesis was that dual time FDG-PET/CT performed at 60 and 180 min after injection of tracer would increase the diagnostic accuracy of FDG-PET/CT imaging (routinely performed at 60 min only) for preoperative assessment of resectability, provided optimal debulking is achievable. Data from 45 patients scanned between 7 Aug 2013 and 7 Jun 2016 were used. The assessment of the FDG-PET/CT scans performed at 60 min was done twice in a blinded fashion with 2 months in between by author MHV in order to investigate the intra-observer repeatability at post imaging processing. The primary ovarian cancer lesion maximum standardised uptake value (SUVmax (g/ml)) was determined when the lesion was identifiable; otherwise, the SUVmax in peritoneal carcinosis was used.

### Software implementation and source code

All analyses were performed by using Stata/MP 14.2 (StataCorp LP, College Station, Texas 77845, USA). The dataset and the Stata source code are accessible as Additional file 1 and Additional file 2, respectively.

## Results

**Fig. 1:**
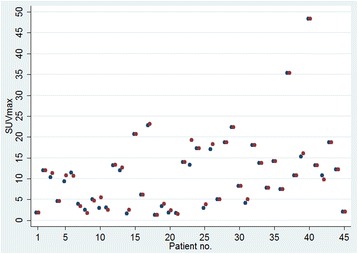
Scatterplot of repeated SUVmax measurements of 60-min scans for 45 patients (blue: 1st reading, red: 2nd reading)

The differences between the two repeated SUVmax readings at 60 min were all less than one in absolute terms, apart from those of patient no. 3, 5, 10, 23, 26, and 42 (Fig. 1). The by far largest difference between the two measurements was observed for patient no. 23 (6 g/ml).

### Bland-Altman limits of agreement

**Fig. 2:**
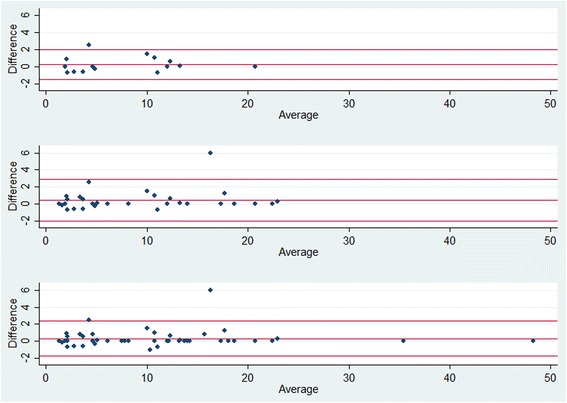
Bland-Altman plots: upper, middle, and lower panel plots comprise *N* = 15, 30, and 45 patients, respectively

At the final analysis (*N* = 45), the estimated mean difference between the paired measurements and the Bland-Altman limits of agreement were 0.30 and − 1.78 to 2.38. Only when patient no. 23 was not part of the first interim analysis was the estimated mean difference smaller and Bland-Altman bands narrower than those at the final analysis (with two interim analyses 0.25, − 1.53 to 2.03 (*N* = 15); with three interim analyses 0.19, − 1.85 to 2.24 (*N* = 11); see Table 1). The estimated mean differences and the Bland-Altman limits of agreement are shown for the analysis strategy comprising two interim analyses (Fig. 2).

**Table 1:** Estimated mean difference and Bland-Altman limits of agreement for the paired differences of measurements

Number of interim analyses	Estimated mean difference and Bland-Altman limits of agreement at analysis time point			
	Interim analysis 1	Interim analysis 2	Interim analysis 3	Final analysis
0	–	–	–	0.30 –1.78 to 2.38 (*N* = 45)
1	0.47 –2.30 to 3.24 (*N* = 23)	–	–	0.30 –1.78 to 2.38 (*N* = 45)
2	0.25 –1.53 to 2.03 (*N* = 15)	0.43 –2.03 to 2.89 (*N* = 30)	–	0.30 –1.78 to 2.38 (*N* = 45)
3	0.19 –1.85 to 2.24 (*N* = 11)	0.47 –2.30 to 3.24 (*N* = 23)	0.40 –1.92 to 2.73 (*N* = 34)	0.30 –1.78 to 2.38 (*N* = 45)

### Fixed-sample analysis (*N* = 45)

Without any interim analysis, no adjustment for multiple testing was necessary and the study was analysed after collection of all data. The observed standard deviation of 1.060 was statistically significantly smaller than that of the benchmark *σ*_0_ = 1.5 (*P* = 0.0022), and the upper confidence limit (uCL) was 1.288, meaning accordingly smaller than 1.5 (Table 2).

**Table 2:** Sequential testing on population standard deviation *σ*

Number of interim analyses	Observed standard deviation, *P* value (sample size), respective significance level, and upper confidence limit at analysis time point			
	Interim analysis 1	Interim analysis 2	Interim analysis 3	Final analysis
0	–	–	–	*SD = 1.060* *P = 0.0022 (N = 45)* *α*_*1*_ *= 0.05* *95% uCL 1.288*
1	SD = 1.415 *P* = 0.3900 (*N* = 23) α_0.5_ = 0.025 97.5% uCL 2.002	–	–	*SD = 1.060* *P = 0.0022 (N = 45)* *α*_*1*_ *= 0.05* *95% uCL 1.288*
2	*SD = 0.909* *P = 0.0162 (N = 15)* *α*_*0.33*_ *= 0.0167* *98.33% uCL 1.495*	SD = 1.255 *P* = 0.1162 (*N* = 30) α_0.67_ = 0.0333 96.67% uCL 1.653	–	*SD = 1.060* *P = 0.0022 (N = 45)* *α*_*1*_ *= 0.05* *95% uCL 1.288*
3	SD = 1.044 *P* = 0.0984 (*N* = 11) α_0.25_ = 0.0125 98.75% uCL 2.006	SD = 1.415 *P* = 0.3900 (*N* = 23) α_0.5_ = 0.025 97.5% uCL 2.002	SD = 1.185 *P* = 0.0452 (*N* = 34) α_0.75_ = 0.0375 96.25% uCL 1.519	*SD = 1.060* *P = 0.0022 (N = 45)* *α*_*1*_ *= 0.05* *95% uCL 1.288*

*SD* standard deviation, *uCL* upper confidence limit, *italics* rejection of null hypothesis

### Sequential testing

Employing one, two, or three interim analyses before the final analysis, statistical testing at all interim analysis time points was adjusted for multiple testing in each strategy by adjusting significance levels as described above. Only the analysis strategy using two interim analyses suggested already sufficient agreement of the repeated readings by rejection of the null hypothesis at the first interim analysis (*N* = 15); the observed standard deviation of 0.909 was statistically significantly smaller than 1.5 (*P* = 0.0162 < *α*_0.33_ = 0.0167), and the uCL was, similarly, 1.495 < 1.5 (Table 2).

Neither incorporation of only one nor implementation of three interim analyses led to early stopping due to sufficient agreement. The influence of one outlier (patient no. 23 in whom the two measurements differed by 6 g/ml) was clearly visible as SD = 1.415 with *N* = 23 exceeded SD = 1.060 at the end of the study (*N* = 45).

## Discussion

### Statement of principal findings

While diagnostic studies in general and agreement studies in particular usually make use of fixed-sample designs, we applied a post hoc group-sequential design for a one-sided hypothesis test setting on the variability of the paired differences in an intra-observer study and exemplified that the conclusions were the same for the first interim analysis (*N* = 15) and the final analysis (*N* = 45) when conducting interim analyses with one third and two thirds of all patients. On the contrary, no interim analysis suggested early stopping when interim analyses were performed with one half or one fourth, one half, and three fourths of all patients due to one influential outlier and our comparably small sample size.

### Strengths and weaknesses of the study

The α-spending function used here [10] is simple and straightforward to apply, and interim analyses open up the opportunity to stop early in case of an early indication of sufficient agreement. Under the assumption of normally distributed differences, the statistical test procedure follows standard theory and has a natural link to the commonly used Bland-Altman limits of agreement.

With small sample sizes as ours, both the testing procedure and the Bland-Altman limits of agreement are sensitive to outliers. Here, the SUVmax assessments for patient no. 23 differed by 6 g/ml. Excluding this patient from the second interim and the final analysis in the analysis strategy comprising two interim analyses led to smaller values for the standard deviation (0.696 and 0.614, respectively) and the upper one-sided 96.67% and 95% confidence limits of *σ* (0.922 and 0.748, respectively), mean differences decreased (0.24 and 0.17, respectively), and Bland-Altman limits of agreement turned narrower (− 1.13 to 1.60 and − 1.03 to 1.37, respectively; data not shown). In general, sample sizes of at least 30 observations are recommended when applying the central limit theorem to the sampling distribution of a mean [8]; when dealing with a variance parameter as target, probably 50 should be the minimum number of paired observations since estimating second moments (like the variance) is more prone to uncertainty than estimating first moments (like the mean).

In order to ensure robustness against single outliers, also interim analyses should comprise ‘sufficiently many’ observations. However, once the analysis strategy is fixed and the time points for interim analyses specified, the investigator needs to stick to the schedule, eventually happily ending with an earlier termination of the study (as in case of our interim analysis with one third of all patients) or having to continue after an early interim analysis due to one outlier (see our alternative partition with the first interim analysis with one half of all patients).

A fundamental challenge in planning an agreement study as the one shown here is the a priori fixation of the hypothesised value for the population standard deviation, *σ*_0_. Moreover, we focused on one simple α-spending function while other less easily accessible α-spending functions gained broader applicability [12, 13]. Finally, we fixed the maximum number of observations of the study and did not consider an adaptive design in which the sample size may be adjusted after the first interim analysis if the original assumptions for the sample size calculations do not hold [14–16].

### Meaning of the study: possible mechanisms and implications for clinicians and policymakers

Agreement studies can either be conducted as such or as part of larger diagnostic accuracy studies for which the assessment of agreement serves quality control purposes; then, usually, just a few sentences are dedicated to agreement results due to limited space for reporting [3]. The employment of group-sequential designs in agreement studies enables early termination when sufficient agreement has been achieved according to an interim analysis. In this way, the image reading extent can be optimised, and resources can be spent more efficiently. The application of group-sequential design methodology in agreement studies should be considered when planning agreement studies in the future.

Group-sequential designs can likewise be easily implemented in other settings than agreement studies. We investigated the diagnostic accuracy of FDG-PET/CT with dual time point imaging (60 and 180 min), contrast-enhanced CT, and bone scintigraphy in patients with suspected breast cancer recurrence previously in a prospective study [17]. Testing the global hypothesis on equality of the areas under the ROC curves was performed once at the end of the study (*N* = 100) but could as well have served as primary hypothesis for interim analyses with, for instance, one half or one third and two third of the sample size. Implementing post hoc group-sequential analysis strategies in the same way as above, i.e. comprising one (at *N* = 50), two (at *N* = 33, 67), or three interim analyses (at *N* = 25, 50, 75), did not lead to early termination of the study at any interim analysis (Table 3). This emphasises the fact that an analysis strategy employing interim analyses may or may not lead to early termination of the study: depending on the effect size and its variability, it may turn out that the originally planned total sample size is still required for demonstration of a statistically significant difference between different regimes.

**Table 3:** Sequential testing on equality of areas under ROC curves [17]

Number of interim analyses	*P* value (sample size) and respective significance level at analysis time point			
	Interim analysis 1	Interim analysis 2	Interim analysis 3	Final analysis
0	–	–	–	0.0189 (*N* = 100) *α*_1_ = 0.05
1	0.1557 (*N* = 50) *α*_0.5_ = 0.025	–	–	0.0189 (*N* = 100) *α*_1_ = 0.05
2	0.0669 (*N* = 33) *α*_0.33_ = 0.0167	0.0577 (*N* = 67) *α*_0.67_ = 0.0333	–	0.0189 (*N* = 100) *α*_1_ = 0.05
3	0.0174 (*N* = 25) *α*_0.25_ = 0.0125	0.1557 (*N* = 50) *α*_0.5_ = 0.025	0.0395 (*N* = 75) *α*_0.75_ = 0.0375	0.0189 (*N* = 100) *α*_1_ = 0.05

### Unanswered questions and future research

How can early stopping rules for both fertility and futility be established in group-sequential agreement studies? Can continuous designs without a priori-specified analysis time points (e.g. triangular test [1]) be adapted to an agreement setting? How can a nonparametric test targeting the spread of data be constructed when the assumption of normally distributed differences does not hold? Can an interrelation be established between such a test and nonparametric Bland-Altman limits of agreement?

## Conclusions

Group-sequential testing in agreement studies offers the possibility of early termination of the trial, implying potential time and resource savings, but timing of and decision rules for interim analyses must be a priori specified in the study protocol in order to secure the experiment-wise type I error probability. Sample sizes must be reasonably large at the time point of interim analysis to ensure robustness against single outliers. Our example was retrospectively analysed, and its results were, indeed, sensitive to one outlier. Group-sequential testing that is widely used in pivotal therapeutic studies of drug development can also be of considerable value in accuracy and agreement studies.

## Additional files

Additional file 1: Dataset from clinical example used. (CSV 1 kb)

Additional file 2: Stata source code for all analyses. (DO 5 kb)

## Abbreviations

CT: Computed tomography
FDG: ^18^F-fluorodeoxyglucose
PET: Positron-emission tomography
ROC: Receiver operating characteristic
SD: Standard deviation
SUVmax: Maximum standardised uptake value
uCL: Upper confidence limit

## References

[1] Whitehead J (1997). The design and analysis of sequential clinical trials.

[2] Moyé LA (2006). Statistical monitoring of clinical trials: fundamentals for investigators.

[3] Kottner J, Audigé L, Brorson S, Donner A, Gajewski BJ, Hróbjartsson A (2011). Guidelines for reporting reliability and agreement studies (GRRAS) were proposed. J Clin Epidemiol.

[4] Zaki R, Bulgiba A, Ismail R, Ismail NA (2012). Statistical methods used to test for agreement of medical instruments measuring continuous variables in method comparison studies: a systematic review. PLoS One.

[5] Altman DG, Bland JM. Measurement in medicine: the analysis of method comparison studies. J Roy Stat Soc D-Sta. 1983;32:307–17.

[6] Bland JM, Altman DG (1986). Statistical methods for assessing agreement between two methods of clinical measurement. Lancet.

[7] Bland JM, Altman DG (1999). Measuring agreement in method comparison studies. Stat Methods Med Res.

[8] Bowerman BL, O’Connell RT, Murphree ES (2016). Business statistics in practice.

[9] Rosner B (2015). Fundamentals of biostatistics.

[10] Kim K, DeMets DL (1987). Design and analysis of group sequential tests based on the type I error spending function. Biometrika.

[11] Gerke O, Vilstrup MH, Segtnan EA, Halekoh U, Høilund-Carlsen PF (2016). How to assess intra- and inter-observer agreement with quantitative PET using variance component analysis: a proposal for standardisation. BMC Med Imaging.

[12] O'Brien PC, Fleming TR (1979). A multiple testing procedure for clinical trials. Biometrics.

[13] Lan GKK, DeMets DL (1983). Discrete sequential boundaries for clinical trials. Biometrika.

[14] Chow SC, Chang M (2012). Adaptive design methods in clinical trials.

[15] Wassmer G, Brannath W (2016). Group sequential and confirmatory adaptive designs in clinical trials.

[16] Dmitrienko A, Tamhane AC, Bretz F (2010). Multiple testing problems in pharmaceutical statistics.

[17] Hildebrandt MG, Gerke O, Baun C, Falch K, Hansen JA, Farahani ZA (2016). [18F] Fluorodeoxyglucose (FDG)-positron emission tomography (PET)/computed tomography (CT) in suspected recurrent breast cancer: a prospective comparative study of dual-time-point FDG-PET/CT, contrast-enhanced CT, and bone scintigraphy. J Clin Oncol.

